# NtGNL1a ARF-GEF acts in endocytosis in tobacco cells

**DOI:** 10.1186/s12870-015-0621-3

**Published:** 2015-11-05

**Authors:** Adriana Jelínková, Karel Müller, Markéta Fílová-Pařezová, Jan Petrášek

**Affiliations:** Institute of Experimental Botany, Academy of Sciences of the Czech Republic, Rozvojová 263, 165 02 Prague 6, Czech Republic; Department of Experimental Plant Biology, Faculty of Science, Charles University in Prague, Viničná 5, 128 44 Prague 2, Czech Republic

**Keywords:** Endocytosis, PIN1 protein trafficking, Inhibitors of endomembrane trafficking, Brefeldin A, Adenosine ribosylation factor (ARF)-guanine exchange factor (GEF), BY-2 tobacco cells

## Abstract

**Background:**

Processes of anterograde and retrograde membrane trafficking play an important role in cellular homeostasis and dynamic rearrangements of the plasma membrane (PM) in all eukaryotes. These processes depend on the activity of adenosine ribosylation factors (ARFs), a family of GTP-binding proteins and their guanine exchange factors (GEFs). However, knowledge on the function and specificity of individual ARF-GEFs for individual steps of membrane trafficking pathways is still limited in plants.

**Results:**

In this work, treatments with various trafficking inhibitors showed that the endocytosis of FM 4–64 is largely dynamin-dependent and relies on proteins containing endocytic tyrosine-based internalization motif and intact cytoskeleton. Interestingly, brefeldin A (BFA), reported previously as an inhibitor of anterograde membrane trafficking in plants, appeared to be the most potent inhibitor of endocytosis in tobacco. In concert with this finding, we demonstrate that the point mutation in the Sec7 domain of the GNOM-LIKE protein1a (NtGNL1a) confers intracellular trafficking pathway-specific BFA resistance. The internalization of FM 4–64 and trafficking of PIN-FORMED1 (PIN1) auxin efflux carrier in BY-2 tobacco cells were studied to reveal the function of the ARF-GEF NtGNL1a in these.

**Conclusions:**

Altogether, our observations uncovered the role of NtGNL1a in endocytosis, including endocytosis of PM proteins (as PIN1 auxin efflux carrier). Moreover these data emphasize the need of careful evaluation of mode of action of non-native inhibitors in various species. In addition, they demonstrate the potential of tobacco BY-2 cells for selective mapping of ARF-GEF-regulated endomembrane trafficking pathways.

**Electronic supplementary material:**

The online version of this article (doi:10.1186/s12870-015-0621-3) contains supplementary material, which is available to authorized users.

## Background

The eukaryotic endomembrane trafficking system is highly dynamic network of organelles that are connected directly or through a system of trafficking vesicles. In higher plants secretory vesicle transport interconnects endoplasmic reticulum (ER), Golgi apparatus (GA), trans-golgi network with the function of early endosome (TGN/EE), endosomal space, prevacuolar compartment (PVC) that later forms the vacuole and plasma membrane (PM) [1, 2]. Budding of vesicles from the donor membrane is accomplished by forming a protein coat responsible for packing of the specific cargo and directing it into its destination. Small guanosine triphosphatase GTPases, such as ARFs, Secretion-associated and Ras-related (SAR) and Secretory (SEC) proteins play essential role in these processes. The switch of GTPase between its active and inactive form is regulated by their GEFs and GAPs (GTPase Activating Proteins) (reviewed by [3, 4]). Clathrin-dependent trafficking, involved in the retrograde and anterograde transport of vesicles in the “post-Golgi” space (between TGN/EE and PM), has been recognized as a major transport in plant cells [5]. The participation of clathrin in the endocytotic internalization of plasma membrane proteins was demonstrated for PIN auxin efflux carriers [6–8]. Besides PINs, internalization of other plasma membrane proteins depend on clathrin, such as the iron transporter IRT1, the boron transporter BOR1, brassinosteroid receptor kinase BRI1, and the aquaporin water channel PIP2 [9, 6, 10–12]. There have been uncovered some players of clathrin-dependent transport in plants as ARF proteins, their GAPs and GEFs (reviewed by [4]). The most studied ARF-GEF GNOM is important regulator of recycling events and with its closest homolog GNOM-like1 (GNL1) protein and ARF-GAP vascular network defective (VAN3) is involved in the selective endocytosis of auxin transport components in *A.t*. [13–15]. The secretory or recycling pathway of the post-Golgi vesicles, designated for exocytosis, ends up by docking and vesicle fusion at the PM with the assistance of SNARE (SNAP (Soluble NSF Attachment Protein) receptor) proteins [16, 17]. Prior to the process of the fusion with the PM vesicles are reversibly tethered to the PM by octameric protein complex, exocyst [18–20].

One of the approaches to study endomembrane transport in vivo is by using inhibitors of various steps of endomembrane trafficking. These drugs affect membrane trafficking by distinct mechanisms. Tyrphostins (TYR), structural analogues of tyrosine are inhibitors of tyrosine kinases and act through their binding to the active sites of the enzymes (reviewed by [2]). Wortmannin (WM) is the inhibitor of phosphoinositol-3-kinase (PI3-kinase) activity [21] and stimulates prevacuolar (PVC)/multivesicular body (MVB) enlargement [22]. Membrane probe filipin (FIL) specifically binds to PM sterols and is often used not only for their detection, but also in higher doses for the inhibition of PM internalization [23, 24]. Dynasore (DNS) is a small molecule that has been reported as highly specific, cell-permeable inhibitor of dynamin GTPase function during clathrin-mediated endocytosis [25, 26]. Also plant hormone auxin was described as an inhibitor of endocytosis in plants [27]. Few studies that address the role of cytoskeleton in the process of endocytosis and protein trafficking in plants use cytoskeletal drugs against actin filaments (AFs) [7, 28–33] and microtubules (MTs) [34]. BFA is a fungal metabolite that is broadly used in studies of vesicle-mediated protein trafficking. It acts as the inhibitor of anterograde protein trafficking, interfering with the function of ARF-GTPases by interacting with their GEFs [35]. Different reaction upon BFA treatment is caused by the sensitivity of individual classes of ARF-GEFs. The sensitivity/resistance of individual ARF-GEFs to BFA is determined by amino acid sequence of their Sec7 domain [13].

In this study, we screened microscopically previously described inhibitors of endomembrane trafficking, auxins and cytoskeletal drugs for their their impact on FM 4–64 endocytosis and PIN1-GFP localization in suspension-cultured tobacco cells. Endocytosis of FM 4–64 appeared to be largely dynamin-dependent, relying on proteins containing tyrosine-based internalization motif and requiring intact cytoskeleton. Suprisingly, in contrast to *Arabidopsis* cells, BFA appeared to be a potent inhibitor of endocytosis of FM 4–64 in tobacco cells. By inserting point mutation into the Sec7 domain of tobacco ARF-GEF *Nt*GNL1a, we have induced its BFA-sensitivity and thus uncovered its preferential role in the endocytosis of FM 4–64 and PIN1.

## Results and discussion

### Characterization of the effects of inhibitors of endomembrane trafficking, auxins and cytoskeletal drugs on the FM 4–64 uptake

**Fig. 1:**
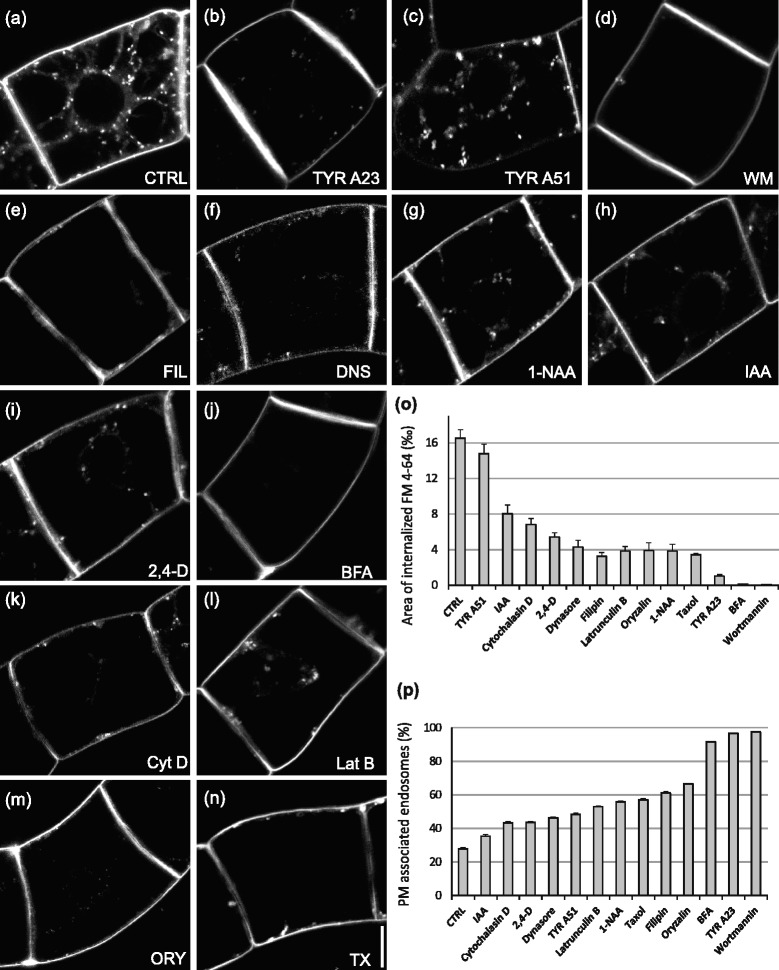
Uptake of FM 4–64 is differently inhibited in the presence of trafficking inhibitors, auxins and cytoskeletal drugs. **a**-**n** In vivo confocal microscopy of FM 4–64 (2 μM, 20 min) uptake by 3-day-old tobacco BY-2 cells pre-treated for 30 min with inhibitors. Confocal sections of perinuclear plane captured with confocal microscope (561 nm excitation). **a** Control (CTRL) cells with mock treatment (DMSO). FM 4–64 endosomes are randomly distributed through the cytoplasm. Note the PM staining. **b** TYR A23 (50 μM) inhibition of the endocytosis in contrast to (**c**) inactive TYR A51 (50 μM), note the absence of the PM staining. **d** WM (33 μM) inhibition of endocytosis. **e** FIL (15 μM), (**f**) DNS (80 μM) and all auxins (**g**) 1- NAA, (**h**) IAA, (**i**) 2,4-D (5 μM) tested, partially inhibiting FM 4–64 uptake. **j** BFA (20 μM) inhibition of endocytosis. **k** Cyt D (40 μM), (**l**) Lat B (500 nm), (**m**) ORY (15 μM) and (**n**) TX (10 μM) partial inhibition of FM 4–64 uptake. Scale bar =10 μm. **o** Relative area of internalized FM 4–64 (expressed in ‰ - per mil of the total cell area) for all inhibitors. Values are means of the ratios between integral areas of the internalized dye (without PM attached endosomes) and total area of the cell. Error bars represent standard error of the means (SEM) from four biological repetitions, *n* = 4. **p** Number of PM attached internalized FM 4–64 endosomes. Values represent the proportion of FM 4–64 endosomes attached to PM to the total internalized FM 4–64. Note the correlation between the amount of cortical (PM attached) endosomes and the rate/inhibition of endocytosis. Error bars represent SEM from four biological repetitions, *n* = 4

The effect of most inhibitors and substances applied in the present study has been previously described, however on distinct plant materials and sometimes with different results. Therefore, we have used FM 4–64 as in vivo endocytic marker and tracked its endocytosis and subsequent distribution within intracellular compartments. Cells were pre-treated 30 min with each inhibitor and the uptake of 2 μM FM 4–64 was observed in interphase cells after 5, 10, 15, 20 and 30 min. Interphase cells with clear nucleoli-containing nuclei were chosen for the analysis, see DIC images in Additional file 1: Figure S2. Mitotic and G1 cells freshly after the cytokinesis were excluded from the analysis, because the rate of endocytosis is enhanced during cytokinesis [36]. From these preliminary experiments ([37] and data not shown), 20 min time point was chosen for all comparisons, where FM 4–64 internalized into discrete endosomes in the cytoplasm (Fig. 1a, Additional file 1: Figure S2a). Integrated area of internalized FM 4–64 was divided by the total area of the cell (defined by PM stained with FM 4–64). Mean values of all ratios (expressed in ‰—per mil of the total cell area) depict the rate of FM 4–64 uptake into the endosomes and other intercellular compartments (Fig. 1o). Moreover, the ratio of PM associated endosomes was quantified (Fig. 1p, Additional file 2: Figure S3). These values helped us to discriminate between inhibition of very early steps of internalization events (for example pinching of the endosome) and inhibition of later endosome intracellular trafficking.

Application of TYR A23 (50 μM) inhibited internalization of the FM 4–64 in comparison with control cells; there were only few FM 4–64 endosomes apparent (Fig. 1b, Additional file 1: Figure S2b). In contrast, TYR A51 (50 μM), previously reported as an inactive tyrphostin with respect to the inhibition of endocytosis [6], did not inhibit the FM 4–64 uptake. More intensive aggregation of the dye and weak PM staining after TYR A51 (Fig. 1c, Additional file 1: Figure S2c) suggest quite rapid FM 4–64 uptake. In *A.t*., TYR A23 has been reported to inhibit the recruitment of endocytic cargo into clathrin-mediated pathway [48].

Specific inhibitory effect of TYR A23 on the PIN2 recruitment, without affecting the FM 4–64 internalization, was previously published in *A.t*. roots [6] as well as in *Arabidopsis* cell suspension culture protoplasts [39]. However, in our experiments with BY-2 suspension cells, TYR A23 clearly inhibited FM 4–64 uptake. These data are in agreement with report of Lam et al. [40], who showed that in BY-2 cells is the effect of TYR A23 dose- and time-dependent. Clear difference between TYR A23 and A51 observed in our work might reflect the fact that endocytic machinery of BY-2 cells has numerous targets for these tyrosine kinase inhibitors or/and that in BY-2 cells the endocytosis of FM 4–64 is dependent on tyrosine-based internalization motif.

Treatment with 33 μM WM resulted in almost complete arrest of FM 4–64 dye uptake at the PM (Fig. 1d, Additional file 1: Figure S2d) and those very small internalized fraction remained in the cortical cytoplasm (Fig. 1p). This is in agreement with already published results [41, 42]. WM has been proposed to be an inhibitor of protein trafficking downstream of the internalization event at the PM [43, 41]. In *A.t*., WM inhibits endocytosis via stabilization of clathrin coated pits formation [5]. It thus can be used as a potent inhibitor of very early steps of endocytosis in BY-2 suspension cells.

**Fig. 2:**
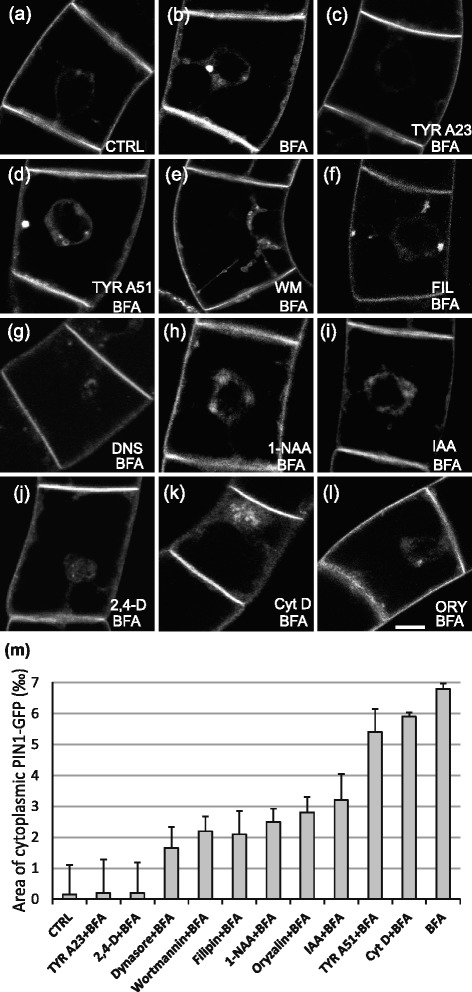
Endomembrane trafficking inhibitors, auxins and cytoskeletal drugs interfere with formation of BFA-induced PIN1-GFP aggregations. **a**-**l** In vivo confocal microscopy of 3-day-old tobacco BY-2 cells transformed with PIN1::PIN1:GFP after 30 min pre-treatment with individual inhibitors, auxins and cytoskeletal drugs followed by 30 min with 20 μM BFA added. Confocal sections of perinuclear plane captured with confocal microscope (488 nm excitation). **a** CTRL cells with mock treatment (DMSO), note PIN1-GFP at the PM. **b** 30 min of BFA (20 μM) treatment inducing intracellular aggregations of PIN1-GFP. **c** TYR A23 (50 μM) preventing the BFA-induced PIN1-GFP intracellular aggregations in contrast to (**d**) TYR A51 (50 μM) pre-treatment. **e** WM (33 μM), note that BFA-induced PIN1-GFP aggregations differ from the BFA effect alone. **f** FIL (15 μM) and (**g**) DNS (80 μM) partially inhibiting the BFA-induced PIN1-GFP intracellular aggregations, note less PIN1-GFP in the cytoplasm. **j** 2,4-D (5 μM) inhibition of BFA-induced PIN1-GFP aggregations (**i**) in contrast to IAA (5 μM) (**j**) and NAA (5 μM) (**h**) with only partial inhibition. **k** Cyt D (40 μM) preventing fusion of BFA-induced PIN1-GFP intracellular aggregations. **l** ORY (15 μM) partial inhibition of BFA-induced PIN1-GFP intracellular aggregations, note less PIN-GFP in the perinuclear area. Scale bar = 10 μm. **m** Relative area of intracellular PIN1-GFP signal after 30 min of BFA treatment alone or with 30 min pre-treatment with inhibitors, auxins and cytoskeletal drugs followed by the addition of 20 μM BFA for 30 (expressed in ‰ - per mil of the total cell area). Values represent the means of the ratios between integral area of the intracellular PIN1-GFP and the total area of the cell. Error bars represent SEM from three biological repetitions, *n* = 3

On the other hand, FIL (15 μM) did not have as dramatic inhibitory effect on FM 4–64 uptake as reported previously in *A.t*. root cells [43], although the trafficking of FM 4–64 to the cytoplasm was inhibited (Fig. 1e, o and p), probably by its binding to PM sterols. DNS (80 μM) treatment partially inhibited internalization of FM 4–64, which stained the PM and adjacent cortical cytoplasm (Fig. 1f). Despite the fact that in plants the clathrin-mediated endocytosis has been proposed [16, 8, 6, 39], the use of DNS in plants has been reported only in tomato as inhibitor of internalization of leucine-rich repeat receptor-like protein2 LeEIX2 [45]. The inhibition of endocytosis with DNS confirmed that the endocytosis of FM 4–64 in tobacco is at least partially dynamin-dependent. To our surprise, the most potent inhibitor of endocytosis in BY-2 was shown to be BFA (20 μM) (Fig. 1j, Additional file 1: Figure S2h, Fig. 1o and Additional file 1: Figure S2i). BFA blocked the endocytosis at the PM, there was almost no FM 4–64 staining visible throughout the cytoplasm and no BFA aggregations or compartments were formed. Auxin was shown previously to inhibit plant endocytosis [27]. When used in the same concentration (5 μM), all three tested auxins, i.e. indole-3-acetic acid (IAA), naphthalene-1-acetic acid (1-NAA) and 2,4-dichlorophenoxyacetic acid (2,4-D) blocked the internalization of FM 4–64 to the roughly same extent (Fig. 1o). However, natively occurring auxin, IAA (Fig. 1h and o) was slightly less effective than synthetic analogues 1-NAA (Fig. 1g, Additional file 1: Figure S2e and Fig. 1o) and 2,4-D (Fig. 1i and o).

To uncover possible role of cytoskeleton in the processes of endocytosis, we also followed effects of cytoskeletal drugs on the uptake of FM 4–64 dye in vivo. Both inhibitors of AFs depolymerisation Cytochalasin D (Cyt D) (20 μM) and AFs polymerization Latrunculin B (Lat B) (0.5 μM) inhibited endocytosis of FM 4–64 (Fig. 1k, Additional file 1: Figure S2h, Fig. 1l and Additional file 1: Figure S2i). Similarly, the inhibition of tubulin polymerization with 15 μM oryzalin (ORY) (Fig. 1m, Additional file 1: Figure S2g, Fig. 1o, Additional file 1: Figure S2i) as well as their stabilization with 10 μM taxol (TX) (Fig. 1n, Additional file 1: Figure S2f, Fig. 1o and Additional file 1: Figure S2i) interrupted the uptake of FM 4–64. Surprisingly, within the literature there is still only limited evidence on the inhibition of FM 4–64 endocytosis after treatments with cytoskeletal drugs in plants. Our data show that that intact cytoskeleton is required for proper progression of endocytosis in BY-2 cells and are in general in agreement with the role of cytoskeleton in plant endocytosis [46].

Altogether, BFA that has been reported previously to inhibit the anterograde membrane trafficking in plants [35], appeared to be the most potent inhibitor of endocytosis in tobacco cells (Fig. 1o and p). Similarly to WM and TYR A23, BFA blocked endocytosis of FM 4–64 at the PM with very few internalized endosomes (Fig. 1o and p), suggesting that these drugs might block more types of endocytic trafficking pathways. BFA-induced inhibition of FM 4–64 uptake into BY-2 cells is in contrast with so far published data by Emans et al. [41] and BFA action in *Arabidopsis* cells, where BFA does not block FM 4–64 uptake neither endocytosis of PIN proteins [13, 23]. Moreover, there are also other cases such as gymnosperm pollen tubes of *Picea meyeri*, where BFA stimulated FM 4–64 uptake [47], which might be explained by the BFA interference with the mechanism of pronounced secretion of cell wall material (polysaccharides, lipids) that is counterbalanced by endocytosis [41]. As Wang et al. [47] showed the exocytosis was inhibited by BFA, while FM 4–64 uptake was 2-fold higher. The exocytosis and thus endocytosis of pollen tubes occurs on the tip of the growing tube. By inhibiting secretion (exocytosis) with BFA, endocytosis may proceed not only at the tip of the pollen tube, but along the whole PM, leading to more disperse signal and enhanced FM 4–64 uptake. Moreover, *Picea meyeri* pollen tubes might contain BFA-sensitive ARF-GEF responsible for the exocytosis and BFA-resistant ARF-GEFs responsible for endocytosis as discussed later.

### BFA-induced intracellular accumulation of PIN1 is differentially triggered by vesicle trafficking inhibitors, auxins and cytoskeletal drugs

Since BFA appeared as potent inhibitor of endocytosis of FM 4–64 (Fig. 1j, Additional file 1: Figure S2h, Fig. 1o and Additional file 1: Figure S2i), it could be suggested that it also stabilizes PM pool of various protein cargoes preventing them from being internalized. We have tested this hypothesis in BY-2 cells transformed with *Arabidopsis* PIN1::PIN1:GFP [48]. As shown in Fig. 2a, in control cells, PIN 1-GFP is located mainly at the PM with only weak signal in the cytoplasm (Fig. 2a and m). After 30 min of 20 μM BFA treatment, dense PIN1-GFP accumulations appeared in the perinuclear area (Fig. 2b). To quantify observed effect, integrated area of internalized PIN1-GFP was divided by the total area of the cell (defined by PIN1-GFP PM staining). Mean values of all ratios (expressed in ‰—per mil of the total cell area) depict the intracellular pool of PIN1-GFP (Fig. 1o).

The pool of PIN1 in these compartments might be both of PM and endomembrane origin, although based on the almost complete inhibition of FM 4–64 endocytosis after BFA shown above, it is more probable that it comes from internal pool of PIN1-GFP. As studied mostly in *A.t*., BFA induces the formation of GA-ER hybrid compartments, so called BFA-compartments [13, 23, 49–51] or loss of Golgi cis-cisternae [52]. The regulation of the amount and positioning of PINs within the PM is achieved by constitutive recycling of PIN-containing vesicles between various PM domains and endosomal compartments [6, 29, 44]. This process is inhibited with BFA, which interferes with the activity of ARF-GEF GNOM. Despite of the fact that GNOM was originally proposed to have plant-specific function in recycling from endosomes to the PM [13], it has been later shown to have additional function in endocytosis [14]. The formation of PIN1-GFP aggregations after BFA observed here further supports our previous results showing decreased auxin efflux in tobacco cells upon treatment with BFA [53].

In addition, the effect of BFA described above allowed us to address the trafficking of PIN1-GFP using all inhibitors applied after BFA pre-treatment. Under these conditions, the FM 4–64-marked internalization is blocked and resulting redistributions of PIN1-GFP might reflect the activity of its internal pool or the fact that for the particular PIN1-GFP pathway the responsible ARF-GEF is BFA-resistant. Indeed, upon BFA pre-treatment, TYR A23 did not induce PIN1-GFP aggregations (Fig. 2c and m), which suggests that besides blocking endocytosis from the PM of FM 4–64 (Fig. 1b, Additional file 1: Figure S2b and Fig. 1o, Additional file 1: Figure S2i) it also blocks formation of BFA-induced PIN1-GFP aggregations from internal pool or PM. In agreement with Dhonukshe et al. [6] and Ortiz-Zapater et al. [39], TYR A51 was inactive (Fig. 2d and m).

After WM treatment, some BFA-induced PIN1-GFP containing aggregations were formed (Fig. 2e and m), but they were morphologically distinct from aggregations induced by BFA alone (Fig. 2b and m). WM caused more massive accumulations of PIN1-GFP in the perinuclear region with smaller aggregations at the cortical cytoplasm (Fig. 2e and m). Since WM also inhibits protein sorting to the vacuole [54] and induces homotypic fusion of MVBs/PVCs [42, 51, 55], we might suggest that these PIN1-GFP aggregates are BFA-induced aggregations originating at TGN/EE and/or MVB/PVC [58]. Moreover, the clear difference between TYR A23 and WM effect on BFA induced PIN1-GFP accumulation (Fig. 2c, e and m) supports our hypothesis on the inhibition of endocytosis of PIN1-GFP from the PM with BFA.

FIL only partially prevented the formation of BFA-induced PIN1-GFP aggregates, there were observed some PIN1-GFP aggregates in the cytoplasm (Fig. 2f and m). DNS blocked the formation of BFA-induced PIN1-GFP aggregates (Fig. 2g and m), suggesting the inhibition of clathrin-dependent trafficking. Since both TYR A23 and DNS block clathrin-dependent transport [25, 38] we could speculate that the formation of intracellular PIN1-GFP accumulations after BFA might be clathrin-dependent.

Both 1-NAA and IAA inhibited the formation of BFA-induced PIN1-GFP aggregations (Fig. 2h, i and m) much less then 2,4-D (Fig. 2j and m). Synthetic auxin 2,4-D is not so good substrate for auxin efflux carrier in contrast to IAA and 1-NAA [56] and thus it is accumulated inside cells, where it could block endocytosis or perhaps even trafficking of intracellular pool of PIN1. The formation of BFA-induced aggregations of PIN1-GFP was not fully prevented by both AFs and MTs drugs. Depolymerisation of AFs with Cyt D resulted in the formation of larger and more diffuse clusters of PIN1-GFP (Fig. 2k). In contrast, MTs depolymerisation with ORY only decreased the amount of aggregations (Fig. 2l and m). These data suggest the involvement of AFs in the trafficking/fusion of PIN1-containing endosomes. The process of translocation of endosomes with PIN proteins from one membrane domain to the other, so-called transcytosis [57] has been shown to involve AFs in the apical-basal targeting of PINs and MTs preferentially in basal targeting [58]. Since there is not clear apical-basal polarity in BY-2 cells, we cannot implicate this model to suspension cells. Previously reported remodelling of AFs in the perinuclear region of tobacco cells by BFA treatment [53] could include active co-operation between AFs and membrane vesicles/endosomes, suggesting the role of AFs in the endosomal fusion.

In summary, BFA-induced PIN1-GFP aggregations are most probably of intracellular origin and their character depends on clathrin-mediated processes, sterol composition of membranes and AFs dynamics.

### BFA action in tobacco cell depends on the composition of ARF-GEFs

**Fig. 3:**
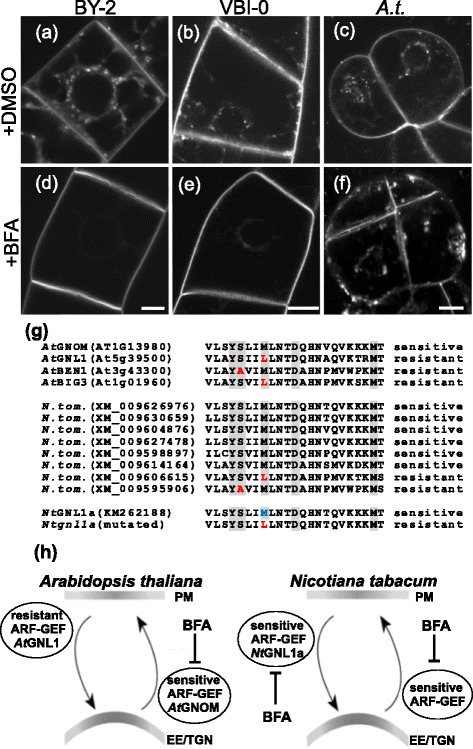
Differential reaction of tobacco and *Arabidopsis* cells to BFA is reflected by the composition of ARF-GEFs. **a**-**n** In vivo confocal microscopy of FM 4–64 (2 μM, 20 min) uptake by 3-day-old tobacco BY-2 and VBI-0 cells and *A.t*. Col-0 cells pre-treated with mock DMSO or 20 μM BFA for 30 min. Confocal sections of perinuclear plane captured with confocal microscope (561 nm excitation). **a**-**c** Control cells with DMSO mock treatment of (**a**) BY-2 (**b**) VBI-0 and (**c**) *A.t*. FM 4–64 stained endosomes are randomly distributed through the cytoplasm. Note the PM staining. **d**-**f** BFA (20 μM)-treated cells. Note the inhibition of FM 4–64 uptake in (**d**) BY-2 and (**e**) VBI-0 cells and PM staining. In contrast, (**f**) *Arabidopsis* cells show no inhibition of FM 4–64 uptake with the accumulated of signal within the cytoplasm. Note the absence of PM staining. Scale bar = 10 μm. **g** Amino acid sequence alignment of the region determining BFA sensitivity/resistance of individual ARF-GEFs (Sec7 domain). *At*GNOM is an example of a BFA-sensitive ARF-GEF, while *At*GNL1, *At*BEN1 and *At*BIG3 are examples of BFA-resistant ARF-GEFs. Residues known to be involved in BFA sensitivity/resistance are highlighted gray and the ones determining resistance are in red. Eight unique Sec7 domains of potential ARF-GEFs in *N. tom*. genome are shown. The “BFA sensitivity” determining methionine residue in *Nt*GNL1a, which was modified to leucine is in blue. **h** Adaptation of the schematic model from Geldner et al. [17] highlighting the role of *N.t*. BFA-sensitive ARF-GEF (*Nt*GNL1a) in ARF-GEF-dependent endocytosis in contrast to the BFA-resistant ARF-GEF (*At*GNL1) in *A.t*.

To address whether observed BFA effect on endocytosis of FM 4–64 in tobacco cells (Fig. 1j, Additional file 1: Figure S2h, Fig. 1o and Additional file 1: Figure S2i) is specie-specific or it reflects the fact that cells are cultured in the artificial form of cell suspension, we compared BFA effect on FM 4–64 uptake in three suspension-cultured cell lines, tobacco BY-2 and VBI-0 and *Arabidopsis* Col-0. In both tobacco cell lines, BFA inhibited FM 4–64 uptake (Fig. 3a, b, d and e). In contrast, in *A.t*. Col-0 suspension cells we observed massive uptake of the dye after 20 min of FM 4–64 incubation and many BFA compartments were formed (Fig. 3c and f).

Since BFA inhibits vesicle trafficking by interfering with the activity of ARF-GEFs [13], it could be predicted that ARF-GEFs responsible for the endocytosis of FM 4–64 are BFA-sensitive in tobacco, but BFA-resistant in *Arabidopsis*. BFA resistance/sensitivity of ARF-GEFs is determined by the amino acid composition of their Sec7 domains. By exchanging specific residues, resistant or sensitive ARF-GEFs might be obtained [13, 59]. BFA-sensitive homolog of GBF, *Nt*GNL1, was cloned from *Nicotiana tabacum* (*N.t*.) [60] and proposed to act at post-Golgi trafficking pathways during embryogenesis, root growth and pollen tube growth [61]. Phylogenetic analysis led to assignment of *Nt*GNL1 to *At*GNL1 class, however, the sequence of its Sec7 domains predicts NtGNL1 to be BFA-sensitive, while AtGNL1 is BFA-resistant [58].

Therefore, we compared amino acid sequence of Sec7 domain of NtGNL1 with all homologous sequences from the recently sequenced and annotated genomes of *Nicotiana sylvestris* (*N.s*.) and *Nicotiana tomentosiformis* (*N. tom*.) [62] using TBLASTN. We found eight unique genes coding for potential ARF-GEFs carrying Sec7 domain in s in *N.s*. and as well as in *N.tom*. genomes. The residues responsible for BFA sensitivity/resistance are in the closest homologous pairs of *N.s*. and *N.tom*. genomes conserved (Additional file 3: Figure S1). Based on the amino acid sequence of Sec7 domain, six potential ARF-GEFs can be predicted to be BFA-sensitive and two of them BFA-resistant (Fig. 3g). In *A.t*., eight ARF-GEFs have been characterized: three members of GBF family: GNOM, GNL1 and GNOM-LIKE2 (GNL2) [63–66] and five members of BFA-inhibited guanine (BIG) family, BIG 1–5 [67–69] exhibiting different sensitivity to BFA [70]. *Arabidopsis* GNOM and BIG1, 3 and 4 are proposed to be BFA-sensitive ARF-GEFs [13, 67, 70]. GNL1, being important for the GA-ER transport [65] and selected endocytosis of auxin transport components [13–15], BIG5 or BFA-visualized ENDOCYTIC TRAFFICKING DEFECTIVE1 (BEN1) being localized into early endosome [69] and BIG3 [67], they all show resistance towards BFA. Moreover, BIG 1–4 ARF GEFs have been recently shown to play a crucial role in post-Golgi trafficking during protein secretion in interphase and cytokinesis. During cytokinesis, BIG1-4 switches its mode and delivers endocytic cargo to the newly formed cell plate [65].

Altogether, it seems that the composition ARF-GEFs is responsible for quite remarkable differences in the overall reaction to BFA between *Arabidopsis* and tobacco cells (Fig. 3h).

### Conversion of BFA-resistant *Nt*GNL1a ARF-GEF to its BFA-sensitive form by site-directed mutagenesis uncovers its role in the endocytosis

**Fig. 4:**
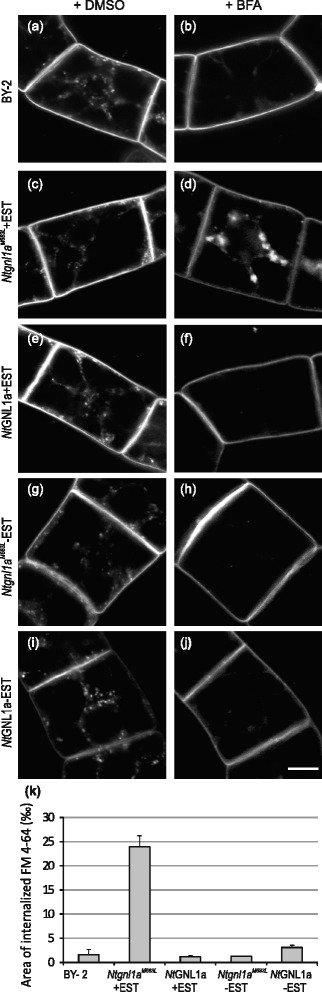
BFA does not block endocytosis of FM 4–64 in BY-2 line upon inducible overexpression of *Ntgnl1a* ^*M683L*^ mutant allele. **a**-**j** In vivo confocal microscopy of FM 4–64 (2 μM, 20 min) uptake by 3-day-old tobacco BY-2 cells, *Ntgnl1a* ^*M683L*^ and *NtGNL1a* cells, non-induced (**g**, **h**, **i**, **j**) and induced with 3 μM β-estradiol (**c**, **d**, **e**, **f**), pre-treatment with mock DMSO (**a**, **c**, **e**, **g**, **i**) or 20 μM BFA (**b**, **d**, **f**, **h**, **j**) for 30 min. Confocal sections of perinuclear plane captured with confocal microscope (561 nm excitation). **a** Control (CTRL) cells with mock treatment (DMSO). FM 4–64-stained endosomes randomly distributed through the cytoplasm. Note the PM staining. **b** BFA (20 μM) inhibition of FM 4–64 uptake in CTRL cells. **c**, **e**, **g**, **i** FM 4–64-stained endosomes randomly distributed in the cytoplasm in induced and non-induced *Ntgnl1a* ^*M683L*^ cells (**c**, **g**) and *NtGNL1a* cells (**e**, **i**). **f**, **h**, **j** BFA (20 μM) inhibition of FM 4–64 uptake in non-induced *Ntgnl1a* ^*M683L*^ cells (**h**) and induced and non-induced *NtGNL1a* cells (**f**, **j**). Note the contrasting uptake of FM–64 in induced *Ntgnl1a* ^*M683L*^ cells (**d**) with BFA-induced FM 4–64 compartments observed in perinuclear area. **k** Relative area of internalized FM 4–64 (expressed in ‰ - per mil of the total cell area). Values represent means of the ratios between integral areas of the internalized FM 4–64 and the total area of the cell. Error bars represent SEM from three biological repetitions, *n* = 3

Since BFA inhibits endocytosis in tobacco cells, we have searched for BFA-sensitive ARF-GEF, which might be responsible for such an inhibitory effect. Amino acid composition of Sec-7 domain of NtGNL1 [60] suggests its sensitivity to BFA, while *At*GNL1 is BFA resistant. Therefore, *NtGNL1* gene was chosen for mutagenesis in Sec-7 domain to replace methionine with leucine at the position 683 [13]. Allotetraploid nature of *N.t*. genome predetermines majority of genes to be presented in two copies inherited from parental genomes, *N.s*. and *N.tom*. During the cloning of *Nt*GNL1 we managed to retrieve its twin sequence and annotated it as *NtGNL1a* (GenBank Acc.nr. KM262188). *NtGNL1a* shares 99 % amino acid identity with its homolog in *N. tom*. (XP_009625271) and 98 % identity with *Nt*GNL1 and *N.s*. form (XP_009789222). We performed site-directed mutagenesis of *NtGNL1a* and generated transgenic tobacco BY-2 lines carrying M683L mutation in *NtGNL1a* under β-estradiol-inducible promoter (*Ntgnl1a*^*M683L*^ line). In parallel, tobacco line carrying wild type *NtGNL1a* gene was generated (*Nt*GNL1a line) and both these lines used for monitoring of FM 4–64 endocytosis in vivo. Interestingly, in contrast to non-induced *Ntgnl1a*^*M683L*^ cells, BFA did not inhibit endocytosis of FM 4–64 in induced *Ntgnl1a*^*M683L*^ cells (Fig. 4c and d), where massive BFA-induced FM 4–64 compartments were observed in the perinuclear area. In both control (Fig. 4b) and non-induced *Ntgnl1a*^*M683L*^ and *NtGNL1a* cells (Fig. 4h and j) as well as in induced *NtGNL1a* cells (Fig. 4f), BFA inhibited the endocytosis of FM 4–64 as expected. The inducible overexpression and transformation themselves did not affect the endocytosis of FM 4–64 (Fig. 4c, e, g, i and k).

**Fig. 5:**
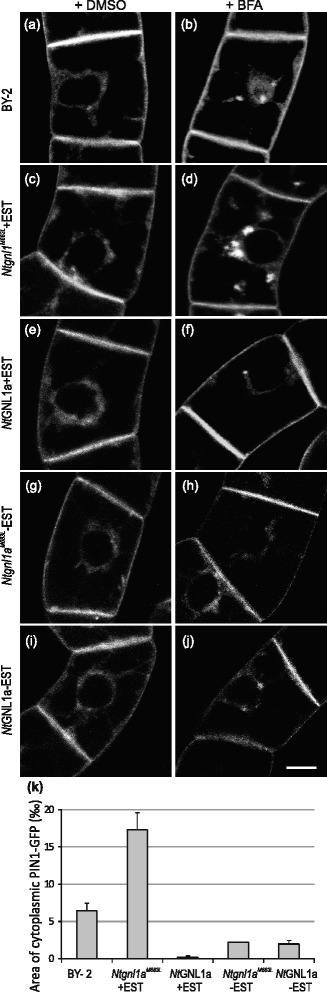
BFA induces massive PIN1-GFP aggregations in cytoplasm in BY-2 line upon inducible overexpression of *Ntgnl1a* ^*M683L*^ mutant allele. **a**-**j** In vivo confocal microscopy of 3-day-old tobacco PIN1::PIN1:GFP cells transformed with *Ntgnl1a* ^*M683L*^ and *NtGNL1a* genes, non-induced (**g**, **h**, **i**, **j**) and induced with 3 μM β-estradiol (**c**, **d**, **e**, **f**), pre-treatment with mock DMSO (**a**, **c**, **e**, **g**, **i**) or 20 μM BFA (**b**, **d**, **f**, **h**, **j**) for 30 min. Confocal sections of perinuclear plane captured with confocal microscope (488 nm excitation). **a** Control (CTRL) cells with mock treatment (DMSO). Note the PIN1-GFP at the PM. **b** BFA (20 μM)-induced aggregations of PIN1-GFP in the perinuclear area. **c**, **e**, **g**, **i** Unchanged PM localization of PIN1-GFP in induced and non-induced *Ntgnl1a* ^*M683L*^ cells (**c**, **g**) and *Nt*GNL1a cells (**e**, **i**). **f**, **h**, **j** BFA (30 min, 20 μM)-induced few PIN1-GFP aggregations in perinuclear area with PIN1-GFP signal remaining at the PM in non-induced *Ntgnl1a* ^*M683L*^ cells (**h**) and induced and non-induced *Nt*GNL1a cells (f, j). Note the contrasting situation in induced *Ntgnl1a* ^*M683L*^ cells (**d**) with BFA-induced FM 4–64 compartments observed in perinuclear area. **k** Relative area of intracellular PIN1-GFP (expressed in ‰ - per mil of the total cell area). Values represent the means of the ratios between integral areas of the intracellular PIN1-GFP and the total area of the cell. Error bars represent SEM from three biological repetitions, *n* = 3

To test whether *N*tGNL1a might be involved in the endocytosis of auxin efflux carrier PIN1, we have transformed *N*tGNL1a and *Nt*gnl1a^M683L^ coding sequence under β-estradiol inducible promoter into PIN1:PIN1:GFP cells [30]. After 30 min of 20 μM BFA treatment, some PIN1-GFP accumulations appeared in the perinuclear area in control (Fig. 5b), in non-induced cells (Fig. 5h and j) as well as in induced *NtGNL1a* cells (Fig. 5f). These clusters might be of intracellular origin (probably TGN/EE, secretory and recycling endosomes), assisted probably by BFA-sensitive ARF-GEFs other than *Nt*GNL1a, as suggested by BFA inhibition of PIN1-GFP endocytosis (Fig. 5f) as well as FM4–64 uptake (Fig. 4j) in induced *NtGNL1a* cells. Less probable explanation is that these BFA-induced clusters are of endocytic origin and PIN1-GFP endocytosis is here assisted by BFA-resistant ARF-GEF in the pathway that is not used for FM 4–64 internalization. Importantly, in induced *Ntgnl1a*^*M683L*^ cells, BFA induced formation of dense and numerous clusters of PIN1-GFP in the perinuclear area (Fig. 5d). The quantification of the area with PIN1-GFP (Fig. 5k) showed that induced *Ntgnl1a*^*M683L*^ cells had dramatically increased amount of internalized PIN1-GFP in comparison with all other samples, the endocytic portion of newly endocytized vesicles was almost triplicate than the endosomal pool (secretory and recycling vesicles, TGN-EE or of GA origin). This suggests that this excessive pool is very probably of PM origin, although significant portion of NtGNL1a might be also active in transient exocytosis events that were reported to occur frequently in regenerating tobacco BY-2 protoplasts [71].

Altogether, these data suggest that BFA-resistant *Nt*GNL1a ARF-GEF may preferentially be involved in the endocytosis of both FM4-64 and PIN1-GFP. Moreover, as schematized in Fig. 3h, the composition of BFA-sensitive and resistant ARF-GEFs determines the extent of BFA effect on the endocytosis, which is interestingly very low in *Arabidopsis* in comparison with tobacco.

## Conclusion

This study describes the identification of tobacco *Nt*GNL1a ARF-GEF and its preferential role in the endocytosis including endocytosis of PM proteins (demonstrated here on auxin efflux carrier PIN1 from *Arabidopsis thaliana* (*A.t*.). It is shown here that the manipulation with the sensitivity of individual ARF-GEFs to BFA can be used as a tool to uncover their pathway-specific functions.

## Methods

### Plant material, gene constructs and transformation

The tobacco BY-2 cell line (*N.t*. L., cv. Bright Yellow-2; [72]) in their exponential growth phase (2–3 days old culture after inoculation), BY-2 cells transformed with *A.t*. PIN1::PIN1:GFP [73, 74] and BY-2 transgenic lines carrying BFA-resistant version of ARF-GEF were cultured in darkness at 27 °C on an orbital incubator (IKA KS501, IKA Labortechnik, http://www.ika.net) at 120 rpm (orbital diameter 30 mm) in liquid medium (3 % sucrose, 4.3 g l^−1^ Murashige and Skoog salts, 100 mg l^−1^ inositol, 1 mg l^−1^ thiamine, 0.2 mg l^−1^ 2,4-D and 200 mg l^−1^ KH_2_PO_4_, pH 5.8) supplemented for transformed cells with 20 mg l^−1^ hygromycin and 100 mg l^−1^ cefotaxim, and sub-cultured weekly. For the gene transformation, basic protocol of An [75] was used. Three-day-old BY-2 cells were co-incubated with *Agrobacterium tumefaciens* [52] strain GV2260 carrying gene constructs in pER8 [76].

VBI-0 tobacco cell strain derived from the stem pith of *N.t*. L., cv. Virginia Bright Italia [77] and *A.t*. cv. Columbia [78] were cultured in standard Heller liquid medium [79] supplemented with synthetic auxins 1-NAA and 2,4-D (5.4 μM, and 4.5 μM, respectively). Cells were sub-cultured every 2 weeks (inoculation density approx.5 · 10^4^ cells ml^−1^) and cultured at 25 °C in darkness on an orbital shaker (INR-200; Sanyo-Gallenkamp, UK) at 150 rpm (diameter 32 mm).

### Chemicals

Styryl dye FM 4–64 (Molecular Probes, catalogue number T13320), BFA, TYR A23, TYR A51, WM, FIL, DNS, cytochalasin D, latrunculin B, ORY, TX and auxins 1-NAA, IAA, 2,4-D (all Sigma-Aldrich) were kept as 20, 50, 50, 50, 10, 20, 80, 10, 20, 57,10 mM and 10, 10 and 10 mM DMSO stock solutions, respectively, and stored in −20 °C.

### Application of FM dyes, inhibitors and auxins

Unless otherwise indicated, the following conditions were used. FM 4–64 (final concentration 2 μM) was added to 1 ml of 2-3-day-old BY-2 cell suspension under continuous shaking in multi-well plates, and samples of interphase cells were observed by confocal microscopy at the times indicated [30]. The inhibitors (TYR A23, TYR A51, WM, FIL, DNS, Cyt D, Lat B, ORY, TX) and auxins (1-NAA, IAA, 2,4-D) were added directly to the cultivation medium to final concentrations of 50, 50, 33, 15, 80, 20,0.5,15,10 μM and 5, 5, 5 μM, respectively for 30 min pre-treatment. 30 min pre-treatment with above-mentioned inhibitors was followed by 30 min concomitant treatment with BFA (20 μM) and analysed by confocal microscopy. The appropriate amount of the solvent (DMSO) was added to controls.

### Cloning and site directed mutagenesis of *NtGNL1a*

Full-length coding sequence of *NtGNL1*a was amplified using cDNA derived from 3-days old BY-2 cell culture as a template. RNA was isolated from 3-days old *N.t*. BY-2 cell culture using RNeasy Plant Mini kit (Qiagen) and reverse transcribed using oligo-dT primers and M-MLV Reverse Transcriptase (Promega). Full-length coding sequence of Nt*GNL1a* was obtained from cDNA derived PCR product using GoTaq Long PCR system (Promega) and 0.25 μM primers (TGU: 5‘-AAC TAT GAT GGG GTG CCT TAA TCA GC-3‘and TGL2: 5‘-GCT TGT GCT TCA ATG AGC GTG TTT CG-3‘, [60]). Amplified fragments were inserted in pGEM-T vector (Promega) and sequenced (Acc.Nr. KM262188). Point mutation (Methionin to Leucin) in the Sec7 domain was performed using site-directed mutagenesis kit (LifeSciences) following manual instructions. Primers for base replacement were: 5’-CGT ATT CAC TTA TCC TGC TGA ACA CGG ATC AAC AC-3’ and 5’-GTG TTG ATC CGT GTT CAG CAG GAT AAG TGA ATA CG-3’. Successful base replacement was confirmed by sequencing and mutated *NtGNL1a* was transferred to plasmid for inducible overexpression in plant cells (pER8, [73]) and used for BY-2 cell culture transformation by *Agrobacterium tumefaciens*.

### Microscopy and image analysis

For the in vivo microscopy, Zeiss LSM 5 DUO confocal microscope with a 40x C-Apochromatic water immersion objective (NA = 1.2) was used. Fluorescence signals for GFP (excitation 488 nm, emission 505–550 nm) and FM 4–64 (excitation 561 nm, emission >575 nm) were detected. All analysis was performed using NIS Elements AR 3.00 (Laboratory Imaging Prague) software. First background fluorescence was subtracted in all images. Next we masked by hand intercellular space (outlined by PM stained with FM 4–64 or PIN1-GFP) of each cell excluding PM attached endosomes.

The integrated area of FM 4–64 or intercellular PIN1-GFP respectively was measured and divided by the total area of the cell. Mean values of all ratios (expressed in ‰—per mil of the total cell area) depict the rate of FM 4–64 uptake (or the intercellular pool of PIN1-GFP) into the endosomes and other intercellular compartments (or intercellular PIN1-GFP distribution).

Number of PM attached internalized FM4–64 endosomes was counted manually. Values represent the proportion of FM 4–64 endosomes attached to PM to the sum of the all endosomes (expressed in %).

All treatments were performed in at least three biological repetitions, for each treatment representative images are shown.

## Additional files

Additional file 1: Figure S2. Uptake of FM 4–64 by 3-day-old tobacco BY-2 cells. Interphase cell in their exponential phase. (a-h) In vivo fluorescence confocal microscopy (upper lane) and Nomarski DIC microscopy (lower lane) after 30 min pre-treatment with inhibitors followed by 20 min staining with 2 μM FM 4–64. Scale bar =20 μm. (i) FM 4–64 internalization quantified using ImageJ software (National Institutes of Health). For each individually analysed cell three values were obtained: mean gray value of the PM, mean gray value of the entire cytosol and mean gray value of the cytosol lacking observable FM 4–64 staining (background intensity). Uptake value of FM 4–64 was calculated as follows: background intensity was subtracted from the mean gray value of the cytosol and the difference was divided by the PM intensity. Uptake values (Ratio of Cytosolic and PM FM-64 mean fluorescence) were normalized to the control. Error bars represent SE, *n* = 40. (PDF 507 kb) Additional file 2: Figure S3. Description of method used for the evaluation of PM associated endosomes. Endosomes close to PM were magnified (green box) and the probe (represented by red line) was applied through the vesicle and the adjacent PM. The pool of PM-associated endosomes was characterized by histogram values that did not decrease below 60 % of PM (endosome) gray values in the region between PM and endosome. The evaluation was performed using ImageJ (National Institutes of Health). (PDF 116 kb) Additional file 3: Figure S1. Sequence alignment of the region determining BFA sensitivity/resistance of individual ARF-GEFs (Sec7 domain) of *N.sylvestris*, *N.tomentosiformis* and *N. tabacum*. Residues known to be involved in BFA sensitivity/resistance are highlighted gray and the ones determining resistance are in red. (PDF 96 kb)

## Abbreviations

1-NAA: Naphthalene-1-acetic acid
2,4-D: 2,4-dichlorophenoxyacetic acid
*A.t*.: *Arabidopsis thaliana*
AFs: Actin filaments
ARFs: Adenosine ribosylation factors
BEN1: BFA-visualized ENDOCYTIC TRAFFICKING DEFECTIVE1
BFA: Brefeldin A
Cyt D: Cytochalasin D
DNS: Dynasore
ER: Endoplasmatic reticulum
FIL: Filipin
GA: Golgi apparatus
GAPs: GTPase Activating Proteins
GEFs: Guanine exchange factors
GNL1: GNOM-like 1
GNL2: GNOM-like 2
IAA: Indole-3-acetic acid
Lat B: Latrunculin B
MTs: Microtubules
MVB: Multivesicular body
*N.s*.: *Nicotiana sylvestris*
*N.t*.: *Nicotiana tabacum*
*N.tom*.: *Nicotiana tomentosiformis*
ORY: Oryzalin
PI3-kinase: Phosphoinositol-3-kinase
PIN1: PIN-FORMED1
PM: Plasma membrane
PVC: Prevacuolar compartment
SAR: Secretion-associated and Ras-related
SEC: Secretory proteins
SNARE: SNAP (Soluble NSF Attachment Protein) receptor
TGN/EE: Trans-golgi network/Early endosome
TX: Taxol
TYR: Tyrphostin
VAN3: Vascular network defective
WM: Wortmannin
