# Supplementary figures and images for: NtGNL1a ARF-GEF acts in endocytosis in tobacco cells
Source: BMC Plant Biol. 2015 Nov 5;15:272. doi: 10.1186/s12870-015-0621-3 (PMC4635988; doi:10.1186/s12870-015-0621-3)

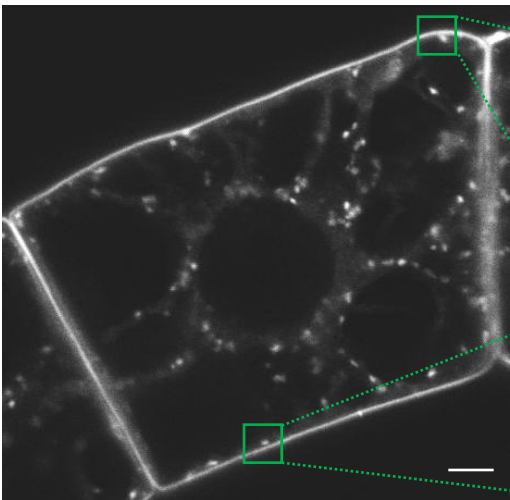

PM-associated vesicle

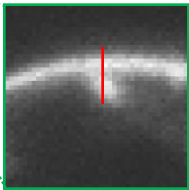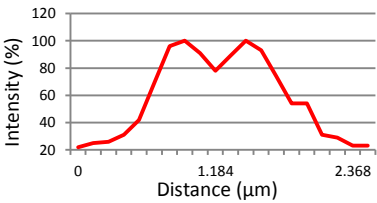

PM-unassociated vesicle

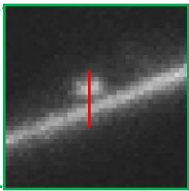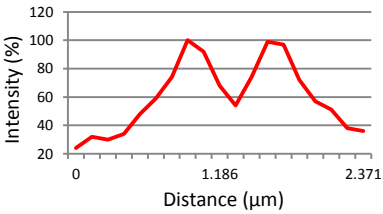

Supplement: Additional file 1: Figure S2. — Uptake of FM 4–64 by 3-day-old tobacco BY-2 cells. Interphase cell in their exponential phase. (a-h) In vivo fluorescence confocal microscopy (upper lane) and Nomarski DIC microscopy (lower lane) after 30 min pre-treatment with inhibitors followed by 20 min staining with 2 μM FM 4–64. Scale bar =20 μm. (i) FM 4–64 internalization quantified using ImageJ software (National Institutes of Health). For each individually analysed cell three values were obtained: mean gray value of the PM, mean gray value of the entire cytosol and mean gray value of the cytosol lacking observable FM 4–64 staining (background intensity). Uptake value of FM 4–64 was calculated as follows: background intensity was subtracted from the mean gray value of the cytosol and the difference was divided by the PM intensity. Uptake values (Ratio of Cytosolic and PM FM-64 mean fluorescence) were normalized to the control. Error bars represent SE, n = 40. (PDF 507 kb) [file 12870_2015_621_MOESM1_ESM.pdf]

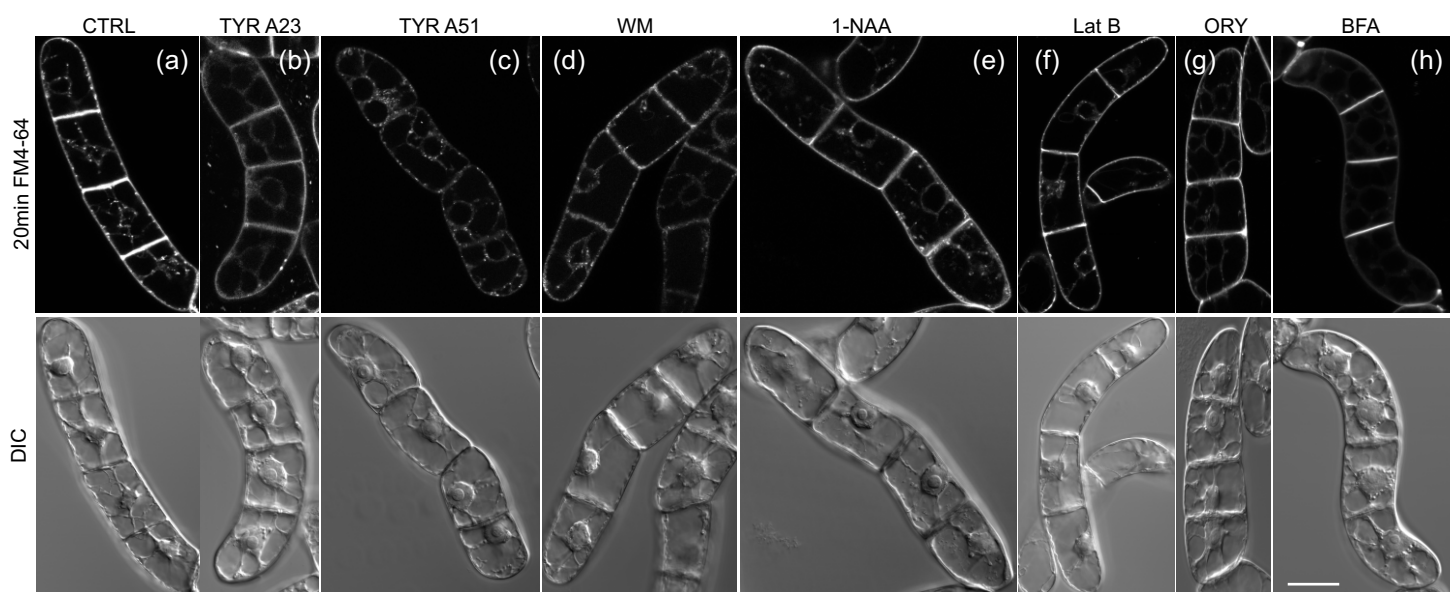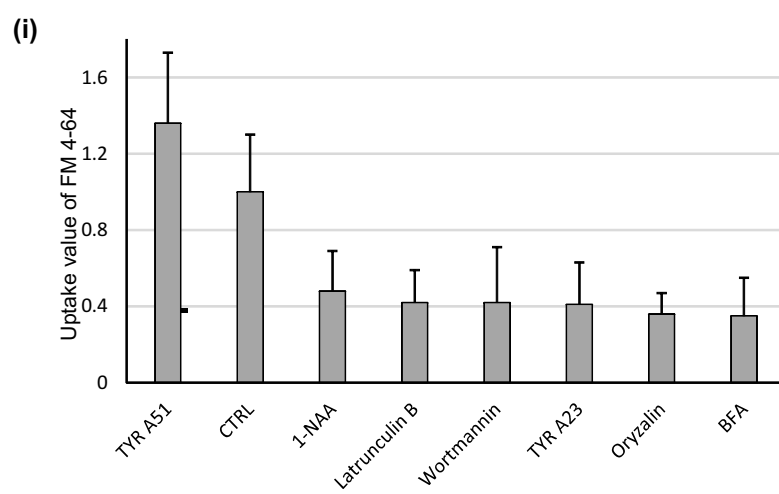

Supplement: Additional file 2: Figure S3. — Description of method used for the evaluation of PM associated endosomes. Endosomes close to PM were magnified (green box) and the probe (represented by red line) was applied through the vesicle and the adjacent PM. The pool of PM-associated endosomes was characterized by histogram values that did not decrease below 60 % of PM (endosome) gray values in the region between PM and endosome. The evaluation was performed using ImageJ (National Institutes of Health). (PDF 116 kb) [file 12870_2015_621_MOESM2_ESM.pdf]
