# Supplementary material for: NtGNL1a ARF-GEF acts in endocytosis in tobacco cells
Source: BMC Plant Biol. 2015 Nov 5;15:272. doi: 10.1186/s12870-015-0621-3 (PMC4635988; doi:10.1186/s12870-015-0621-3)
Supplement: Additional file 3: Figure S1. — Sequence alignment of the region determining BFA sensitivity/resistance of individual ARF-GEFs (Sec7 domain) of N.sylvestris, N.tomentosiformis and N. tabacum. Residues known to be involved in BFA sensitivity/resistance are highlighted gray and the ones determining resistance are in red. (PDF 96 kb) [file 12870_2015_621_MOESM3_ESM.pdf]

Organism	GenBank Acc.Nr.	Sec7 sequence alignment	BFA-sens./res.
NtGNL1a	(KM262188)	VLSYSLIMLNTDQHNTQVKKKMT	sensitive
NtGNL1a	(EF520731)	VLSYSLIMLNTDQHNTQVKKKMT	sensitive
N.tom.	(XM_009626976)	VLSYSLIMLNTDQHNTQVKKKMT	sensitive
N.s.	(XM_009790920)	VLSYSLIMLNTDQHNTQVKKKMT	sensitive
N.tom.	(XM_009630659)	LLSYSLIMLNTDQHNTQVKKKMT	sensitive
N.s.	(XM_009791036)	LLSYSLIMLNTDQHNTQVKKKMT	sensitive
N.tom.	(XM_009604876)	VLSYSVIMLNTDQHNVQVKKKMT	sensitive
N.s.	(XM_009762979)	VLSYSVIMLNTDQHNVQVKKKMT	sensitive
N.tom.	(XM_009627478)	LLSYSIIMLNTDQHNVQVKKKMT	sensitive
N.s.	(XM_009791968)	LLSYSIIMLNTDQHNVQVKKKMT	sensitive
N.tom.	(XM_009598897)	ILCYSVIMLNTDQHNPQVKKKMT	sensitive
N.s.	(XM_009790966)	ILCYSVIMLNTDHHNPQVKKKMT	sensitive
N.tom.	(XM_009614164)	VLAYSVIMLNTDAHNSMVKDKMT	sensitive
N.s.	(XM_009796667)	VLAYSVIMLNTDAHNSMVKDKMT	sensitive
N.tom.	(XM_009606615)	VLAYSVILLNTDAHNPMVKTKMS	resistant
N.s.	(XM_009800792)	VLAYSVILLNTDAHNPMVKTKMS	resistant
N.tom.	(XM_009595906)	VLAYAVIMLNTDAHNPMVWPKMS	resistant
N.s.	(XM_009801996)	VLAYAVIMLNTDAHNPMVWPKMS	resistant
